# Interfaces between Biological Theory of Human Development and DIR/Floortime in the understanding and treatment of autism

**DOI:** 10.1192/j.eurpsy.2023.1558

**Published:** 2023-07-19

**Authors:** P. M. Pacheco, M. D. S. Pacheco, D. R. Molini-Avejonas

**Affiliations:** 1 Speech Language Pathology, University of São Paulo; 2 Morphology, Federal University of Espirito Santo, Vila Velha; 3 Speech Language Pathology, University of São Paulo, São Paulo, Brazil

## Abstract

**Introduction:**

Autism can be described as a mental disorder that displays social interactions and communication impairments as well as a restricted range of activities or interests. Since autism is different for each individual, possible treatments are challenging and should consider individual characteristics at all times. Interactions with peers, family, and teachers are challenging for those with autism as they usually lack behaviors such as eye contact, playing, and talking with other people. It is common to observe sensorial issues as hypersensitivity in these individuals. Patients may have visual, auditive, or even tactile dysfunctions.

Dir/Floortime is a comprehensive model that gives theoretical support and methodological approach to lead to development focusing on the development of individual capacities for sensorial organization, motor planning, language, and many abilities that provide a development trail that will help individuals to achieve essential milestones to infants and adolescents. The DIR/Floortime is based on the development as a lifespan event, individual differences, and relationships established with peers and any other people in the child´s context.

**Objectives:**

The aim of this study was to demonstrate that the DIR/Floortime is a comprehensive method of study and intervention since it matches all necessary characteristics to produce development.

**Methods:**

It was performed a theoretic approach of both DIR/Floortime and Biological Theory of Human Development in search of basis in a contextualist theory to explain a practical method of intervention.

**Results:**

Psychological theories about human development are important tools for understanding the way individuals interact with their context and produce changes in biopsychosocial characteristics. The Bioecological theory of Human Development is a contextualist theory that considers the interactions established between individuals through a model called PPCT, with their characteristics with people, objects, and symbols through interactions known as proximal processes, considered to be meaningful interactions, occurring frequently, through a long time.

**Conclusions:**

The TBDH through the PPCT model can show that the DIR/Floortime presented itself as an efficient method for the treatment of autism since it considers the personal characteristics of the patients, especially their sensory and motor characteristics (Personal Characteristics such as Resource, Demand, and Force). This method has in significant interaction the most efficient way to produce development (equivalent to proximal Proximal Processes), takes into account the contexts where the developing individual attends, especially home, school and therapy, and has time as an essential factor for development to occur.

**Disclosure of Interest:**

None Declared

